# Statistical Characterization of Temperature and Pressure Vertical Profiles for the Analysis of Laser Heterodyne Radiometry Data

**DOI:** 10.3390/s21165421

**Published:** 2021-08-11

**Authors:** Monica M. Flores, David S. Bomse, J. Houston Miller

**Affiliations:** 1 Department of Chemistry, George Washington University, Washington, DC 200521, USA; mmflores@email.gwu.edu; 2 Mesa Photonics, 1550 Pacheco Street, Santa Fe, NM 87505, USA; dbomse@mesaphotonics.com

**Keywords:** laser heterodyne radiometry, remote sensing, greenhouse gases

## Abstract

The statistical analysis of historic pressure and temperature profiles from radiosonde launches for use in the fitting of molecular oxygen line shapes is presented. As the O_2_ mixing ratio is nearly constant throughout the lower atmosphere, only variations in pressure and temperature profiles will affect the fit of observed O_2_ features in Laser Heterodyne Radiometry (LHR) spectra. Radiosonde temperature and pressure data are extracted from the Integrated Global Radiosonde Archive (IGRA) for a given station, date, and launch time. Data may be extracted for a single launch, for the same date over several years, and/or within a window centered on a target date. The temperature and pressure profiles are further characterized by the statistical variation in coefficients of polynomial fits in altitude. The properties of the probability distributions for each coefficient are used to constrain fits of O_2_ line shapes through Nelder–Mead optimization. The refined temperature and pressure profiles are then used in the retrieval of vertically resolved mixing ratios for greenhouse gases (GHGs) measured in the same instrument. In continuous collections, each vertical profile determination may be treated as a Bayesian prior to inform subsequent measurements and provide an estimate of uncertainties.

## 1. Introduction

The determination of precise greenhouse gas (GHG) mixing ratios in the lower troposphere, where air is less well-mixed than at higher altitudes, is essential to pinpointing sources of pollution and trace gas species. Currently, vertical profile measurements of GHGs include weather balloons (e.g., radiosondes), aircraft, Low Earth Orbiting (LEO) satellites such as NASA’s OCO-2 and JAXA’s GOSAT [1,2], and ground-based measurements such as Laser Heterodyne Radiometry (LHR). LHR measurements interrogate the full atmospheric column from the ground using the Sun as a spectroscopic light source. Compared with satellite-based instruments, ground-based LHR is less expensive, avoids pathlength ambiguities due to backscattered sunlight, eliminates near-surface pressure uncertainties, and has better response toward gases in the lower troposphere.

LHR absorption peaks are the line-of-sight, summed contributions from gases throughout the full atmospheric columns. Absorption line shapes vary with altitude due primarily to changing pressure and, less significantly, to varying temperature and collision partners. The quality of LHR spectra, particularly at resolutions ≤200 MHz (≤0.007 cm^−1^), facilitates non-linear deconvolutions—retrievals—that extract gas concentration vertical profiles from ground-based data. Retrieval precision can be improved by including LHR spectra of O_2_ because it has a nearly uniform concentration throughout the troposphere and lower stratosphere. As a result, O_2_ LHR spectra are dominated by pressure and temperature effects, allowing retrieval algorithms to emphasize determining those two parameters that can then be folded back into GHG and water vapor retrievals.

Errors in atmospheric transport models currently applied to atmospheric measurements limit the accuracy of GHG fluxes. Ground-based vertical profile measurements are less subject to vertical transport than satellite measurements, making them more useful in constraining surface flux determinations. Temperature trends are closely related to the tropospheric lapse rate and atmospheric water vapor content, amplifying the need for accurate models of climate feedback processes. Due to these complex, interconnected feedback mechanisms concerning GHGs, the simultaneous measurement of target species provides the precise data needed for weather forecasting and climate modeling.

One of the most commonly used methods for the retrieval of mixing ratios from LHR data is the optimal estimation method (OEM) developed by C. D. Rodgers, to which vertical profiles can be fed from sources such as European Centre for Medium-Range Weather Forecasts (ECMWF) or National Centers for Environmental Prediction (NCEP) [3,4,5,6]. Weidmann et al. demonstrated the application of this method to LHR data using pressure, temperature, and volume mixing ratios extracted from ECMWF [7]. While ECMWF and NCEP are commonly used sources for vertical profile parameters, in the case of Schneising et al., initial guesses for vertical mixing fractions, pressure, and temperature for GHG dry air mole fraction determination are based on the U.S. Model Standard Atmosphere, 1976 [8].

A recent derivative of the OEM method that has been presented for LHR data processing makes use of the Planetary Spectrum Generator (PSG) API to extract Modern Era Retrospective Analysis, Version 2 (MERRA-2) vertical profiles a priori for the spectral fitting routine [9]. Spectral simulations using pressure, temperature, and volume mixing ratios from MERRA-2 are used to calculate spectra by fitting CO_2_ abundances using the OEM. MERRA-2 data are based on satellite reanalysis data and provide several product options with data ranging from monthly to hourly averages. A limitation of this database is that data availability is limited by a lag of several days up to one month for certain products. Palmer et al. use the M2I3NVASM component, which does not update daily, severely hindering the capability for near-real time data analysis [9].

Radiosondes are reliable instruments that have been used to correct biases in satellite measurements as well as to provide validation and cross-comparison of planetary boundary layer (PBL) and precipitable water vapor (PWV) determinations, upper troposphere and atmospheric temperature model analyses, and cloud-affected radiance modeling [10,11,12,13,14]. In addition to being established instruments, radiosondes provide better vertical resolution than the satellite data used in reanalyses [15], allowing for a clearer differentiation in temperature and water trends with varying altitude. For this reason, in this study we present a statistical characterization method for the analysis of historic pressure and temperature vertical profiles from radiosonde data obtained from the Integrated Global Radiosonde Archive (IGRA) to inform LHR O_2_ line shape spectral fitting [16]. This method is then applied to data analysis from our LHR derivative, Precision Heterodyne Oxygen-Corrected Spectroscopy (PHOCS) instrument being developed with the goal of producing an autonomous, cost-effective GHG monitoring system [17].

## 2. Methods

### 2.1. Experimental Details

#### LHR Instrumentation and Data Retrieval Method

Mesa Photonics and George Washington University are developing a variant of an LHR known as PHOCS that simultaneously collects high-resolution oxygen spectral line shape data and target species spectra. Our LHR instrument is comprised of two main units, detailed in a recent publication—the electronics chassis and the sun tracker system [17]. In the first generation of this instrument, the electronics chassis houses a 1278 nm laser for O_2_ and H_2_O and a 1572 nm laser for CO_2_ measurements. In the second-generation instrument, a 1651 nm laser for CH_4_ measurements will also be included in the electronics chassis.

For each laser wavelength region, transmission spectra are collected as heterodyne rf power. Sensor GPS location, sun angle, and time of day are also recorded. The IGRA pressure and temperature profiles as a function of altitude, described below, may then be used to refine spectral fitting parameters first for the 1278 nm, O_2_ and H_2_O, spectrum and then for the 1572 nm, CO_2_, spectrum. A long-term goal for PHOCS is for it to be a reliable, fully automated system for atmospheric profiling in which gas concentrations retrieved in real-time can be folded back in as Bayesian priors, driving our need for near real-time pressure and temperature profiles.

As oxygen concentrations in the troposphere and lower stratosphere are nearly constant, these line shapes are uniquely sensitive to both temperature and pressure profiles, and constrained fitting of these line shapes enables more precise GHG concentration retrievals. Concentration retrieval of target species (e.g., H_2_O, CO_2_, and CH_4_) relies on both dry air corrections from oxygen measurements as well as accurate profiles of pressure and temperature through the troposphere and lower stratosphere. These latter quantities can also be derived from accurate and precise oxygen spectral feature modeling.

In order to obtain accurate atmospheric models, spectroscopic parameters for the gases of interest must be known, including the temperature and pressure-dependent broadening of the half-width of spectral lines. The temperature dependence of the half-width is known to have a power-law form, and recently a double power law has been investigated to improve fits of half-width with respect to varying temperature [18]. As temperature on a hot summer day can range from about 37 °C at the surface to −51 °C at the tropopause and −40 °C in the lower stratosphere, temperature effects on line widths must be taken into account for spectral fitting. This demonstrates the need for refined pressure and temperature fit parameters to obtain precise O_2_ concentrations.

### 2.2. Integrated Global Radiosonde Archive Network

With over 2700 stations worldwide, the global coverage for radiosondes is extensive and provides more precise vertical resolutionthan satellite data from the surface level to ~35 km in altitude (very few balloons survive higher than this altitude). Radiosonde stations, such as those as part of the National Weather Stations (NWS) network, are launched twice daily at 00:00 and 12:00 UTC. Due to the accessibility of radiosonde data on a fast timescale, they can be used for cross-comparison and calibration of other instrumentation.

#### Measurement Sites and RS Station Locations

Work to date has centered on launches from Sterling, Virginia (Station ID: USM00072403, Latitude: 37.9333, Longitude: −77.4858), located near Dulles International Airport (IAD), which was chosen due to its proximity to the measurement field sites in and around Washington, D.C. (GW campus building SEH, Smithsonian Environmental Research Center SERC, and Nellysford, VA, USA).

### 2.3. Radiosonde Data Statistical Analysis Method

#### 2.3.1. Overview of Temperature and Pressure Vertical Profile Retrieval

As the O_2_ mixing ratio is nearly constant throughout the lower atmosphere, only variations in pressure and temperature profiles will affect the shape of observed O_2_ spectral features. Our goal in this work is to constrain these profiles through the consideration of relevant radiosonde data. As noted above, vertical pressure and temperature profiles may be obtained from standard atmosphere models and reanalysis datasets such as those provided by ECMWF [19]. However, standard atmosphere estimates do not capture the dynamic shape and height of the tropopause and may contain discontinuities compromising their utility as starting estimates in fitting of the O_2_ spectral feature. Another source for temperature profiles is IGRA. By using fit coefficients constrained by radiosonde data, the vertically resolved pressure and temperature profiles can be determined in the fit of O_2_ features.

#### 2.3.2. Radiosonde Data Retrieval and Statistical Analysis

**Figure 1:**
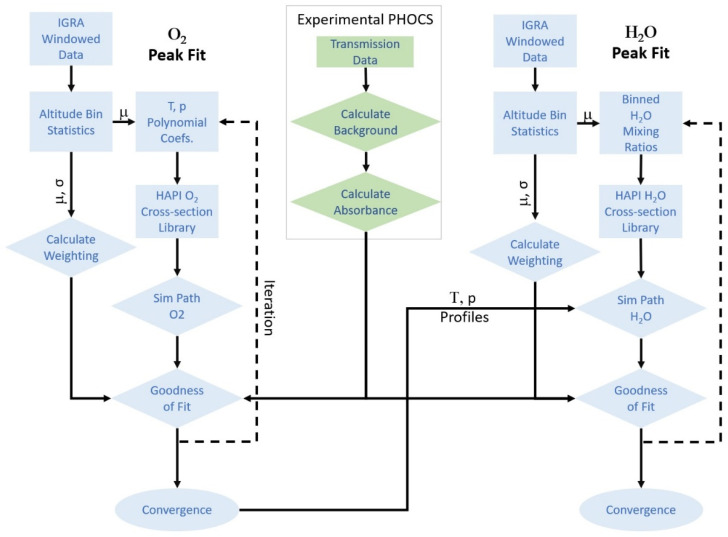
An illustration of the RS and PHOCS data extraction and analysis method. The PHOCS instrument stores raw heterodyne power as well as meta data for a given measurement. From this raw data, a background spectrum is calculated from spectral points removed from absorption features, and an absorption spectrum is generated. Informed by the meta data, IGRA data is collected from nearby stations, sorted into altitude bins, and statistical calculations for that windowed set are calculated. Polynomial fits of temperature and pressure are performed from the mean of each bin. Spectra for the atmospheric column are then simulated by calculating contributions from each 100 m path along this path by calling spectral cross sections from pre-calculated libraries. Weighted residuals between modeled and experimental spectra are calculated and iterated in a Nelder-Mead algorithm until convergence.

A radiosonde extraction program developed at GWU accesses National Oceanic and Atmospheric Administration (NOAA) radiosonde data via the IGRA webpage for a given station, date, and launch time. The program can extract single launch data, data for the same date over several years (typically the last decade is used), and/or data within a window centered on a target date. The temperature and pressure are further evaluated to extract statistical data for the spectral fitting code. A summary of this process is shown in Figure 1.

## 3. Data and Results

### 3.1. Statistics Obtained for PHOCS Measurements: 29 July 2019

**Figure 2:**
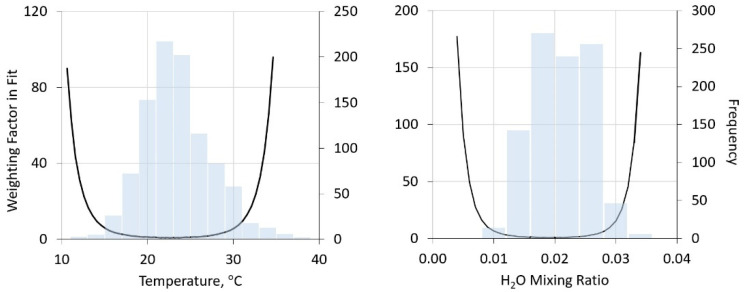
Frequency distribution of lowest altitude (surface to 1 km) temperatures (**left**) and water mixing ratio (**right**) in Dulles airport radiosonde launches (grey bars) and the derived “weighting factor” for fitting, as defined in Section 3.1.3 below.

For this preliminary analysis, historic radiosonde data were analyzed for measurements taken in the mid-Atlantic region of the United States for select days in summer 2019. RS data were extracted from the Dulles station in the date range of 25–31 July 2010–2020 to have a 7-day window of historical data centered on the measurement date. Data for each launch are sorted into 1 km altitude bins. For each binned set, statistics are calculated including mean, standard deviations, and skewness. As an example, Figure 2 shows statistics for temperature within the lowest bin (surface to 1 km) in the Dulles airport launches. The skewness for the first bin is somewhat larger than those observed at higher elevations, perhaps attributable to the exposure of the launch package prior to launch. However, in general the binned data are symmetric and well described by normal distributions.

#### 3.1.1. Pressure

**Figure 3:**
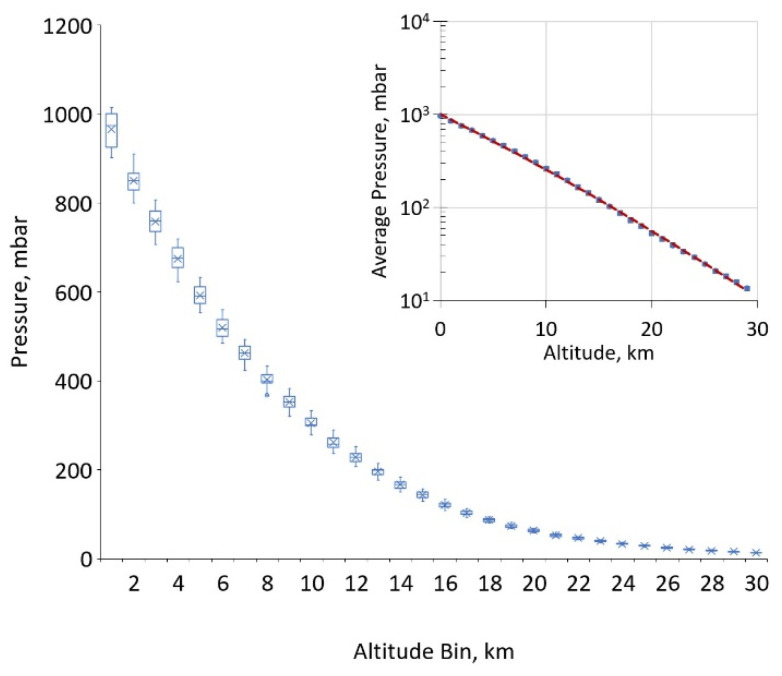
The extracted pressure profiles for the decade-long, 7-day window are sorted into 1 km bins to visualize pressure distributions at different altitudes. Note: the statistical spread for pressure is not discernible in the inset, logarithmic plot.

Pressure has a near exponential decay with altitude and is well described by a 2nd-order polynomial fit of the log of pressure, as shown in Figure 3. Box and whisker plots are constructed to explore probability distribution functions (PDFs) for the coefficients and to explore the occurrence of and importance of outliers. These PDFs are then used to establish weighting parameters for each pressure-fitting coefficient in the PHOCS fitting program for oxygen line shapes. The pressure PDFs are used to estimate uncertainties in pressure profiles and thus propagate to uncertainties in derived GHG concentrations for different atmospheric levels.

#### 3.1.2. Temperature and Water

**Figure 4:**
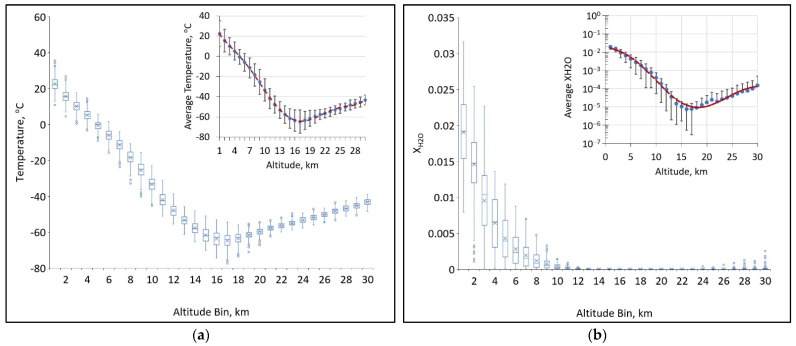
Extracted temperature and water profiles sorted into 1 km bins to visualize distributions at different altitudes. The insets show (**a**) average temperature and (**b**) average log_10_(X_H2O_) with 1σ deviations shown as vertical bars and 6th order polynomial fits for data over the decade-long, 7-day window.

A similar protocol was used for temperature and water profiles derived from radiosonde measurements where data are binned into 1 km intervals and statistical parameters are extracted for each bin, shown in Figure 4 (water profiles are used to inform dry air corrections of GHG mixing ratios).

Both the temperature and water profiles have more complex vertical behavior than that of pressure. For spectral fitting, either may be characterized by a 6th-order polynomial fit of mean bin values over the decade-long, 7-day window. The binning of temperature and water vapor data allows us to constrain errors in tropopause temperatures, allowing for a better fit in this region of the atmosphere. While both distributions are nearly Gaussian within each 1 km interval, water has considerably larger standard deviations throughout the troposphere, particularly at the lowest altitudes.

#### 3.1.3. Temperature and Pressure Retrieval Procedure

The core of our LHR modeling and line fitting has been described in prior publications [17,20]. In brief, atmospheric spectra are simulated for the column using a spectral simulation package developed at the George Washington University. This software uses physical parameters from the HITRAN spectral database to model spectra using components of the HITRAN Application Programming Interface (HAPI) [21,22]. Specifically, molecular absorption cross sections are calculated for each spectral window at 0.001 cm^−1^ resolution for every combination of atmospheric pressure and temperature at 1 mbar and 1 K resolutions, respectively (from 1 to 1100 mbar and 200 to 310 K). These simulations are performed using Voigt line shapes calculated by the HAPI [22]. The implementation of speed-dependent Voigt line shapes is the subject of on-going work in our group using recently available parameters [23] for oxygen features. Data are stored in indexed, binary files for later use. Integrated path absorption spectra are then calculated using the initial sun angle and pressure and temperature profiles to calculate target molecule densities. As noted above, there are three fitting variables to characterize pressure and seven for temperature. In addition, an eleventh fitting parameter is used to provide for any drift in laser wavelength. This fitting parameter is constrained to not exceed ±0.01 cm^−1^ but in practice has been found to be <±0.003 cm^−1^, which is approximately half of the first-generation instrument’s resolution.

For each atmospheric level, at each step in the Nelder–Mead optimization, eleven parameters are allowed to vary to produce temperature (6th order polynomial) and pressure (2nd-order polynomial) profiles; a spectral simulation is performed; and a goodness of fit is constructed from the sum of squares of residuals between modeled and observed spectra for each point across the oxygen feature. These residual sums are then multiplied by a weighting factor, *W*, calculated for the temperature and pressure coefficients as:

(1) $$W\;=\;\sum_{i=1}^{10}\left[\sum_{i=j}^{30}\frac{1}{e^{-\frac{1}{2}{\left(\frac{x-\mu_{i,j}}{\sigma_{i,j}}\right)}^{2}}}\right]$$

where the outer sum in *i* is over the total of 10 temperature and pressure coefficients and the inner sum in *j* is over each of the 30 atmospheric layers, or bins. *x* refers to the temperature or pressure calculated at the midpoint for a particular altitude bin; *μ* refers to the mean for the scalar within that bin; and *σ* refers to the standard deviation in that quality for that bin. By increasing the magnitude through weighting, distant “outliers”—more than 3*σ* from the mean—are penalized (for example, a mid-summer, surface temperature of 5 °C in the Mid-Atlantic region of the U.S. is highly unlikely).

#### 3.1.4. Line Fitting, Temperature and Pressure Profile Results

**Figure 5:**
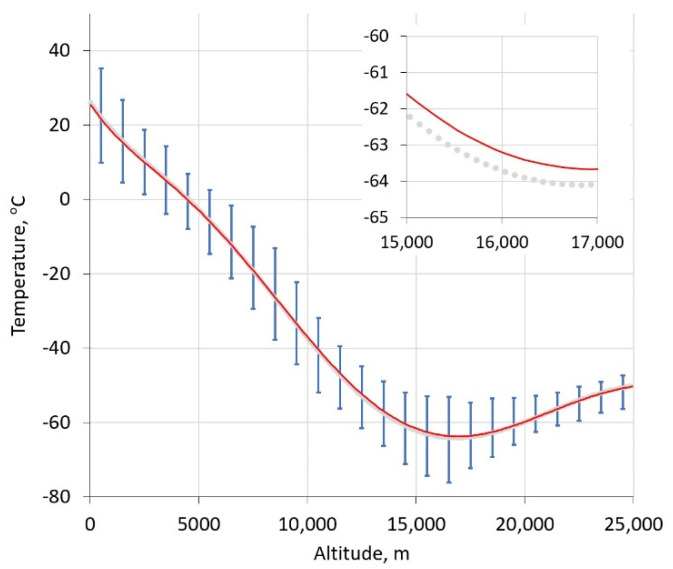
Temperature profiles before (grey symbols) and after (red) fit of oxygen line shape. The inset shows the region surrounding the tropopause, emphasizing the small difference in initial and final profiles for this spectrum. Vertical lines indicate the full span of observed radiosonde data within each altitude bin.

**Figure 6:**
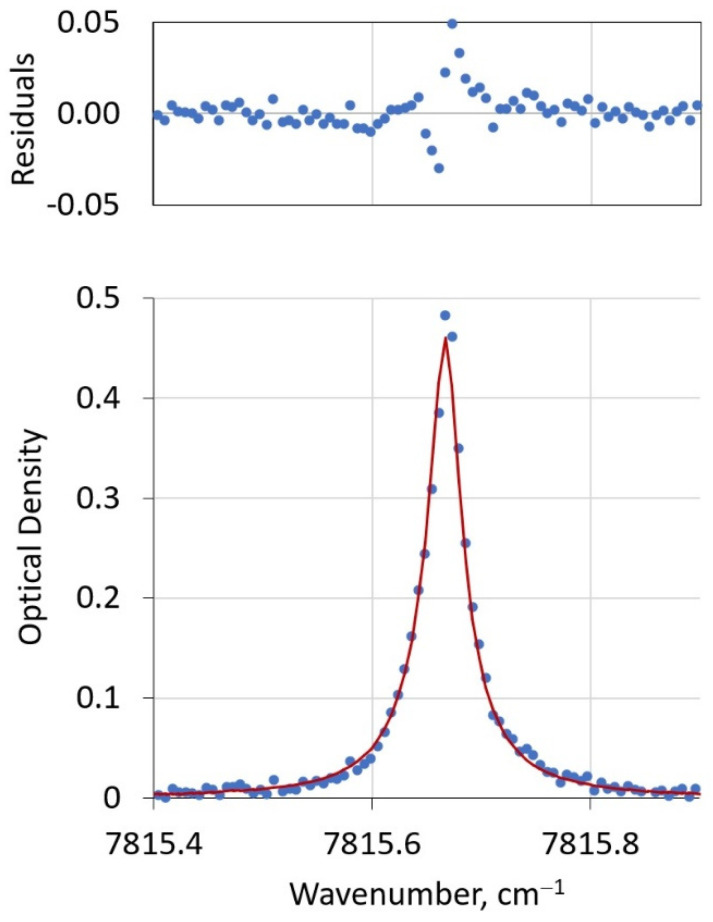
Lower panel: representative fit of PHOCS oxygen line. Experimental data are shown as blue symbols. The red solid curve is the modeled spectrum. The upper panel shows the residuals of the fit. The instrument resolution, 200 MHz or 0.067 cm^−1^, is the spacing between experimental (blue) points.

Figure 5 shows an example temperature profile before (from the mean of the binned, historic, radiosonde data) and after optimization in the spectral fitting. For this fit, these profiles showed little change during the spectrum optimization, perhaps owing to the quality of the initial spectrum shown in Figure 6, but also a reflection of the relatively tight distributions in the binned temperature data. On average, the full span in observed readings was found to fall within ±2.7 standard deviations across all altitudes. The residuals of the fit show the largest deviations near the line center, which we feel is attributable to the current 200 MHz instrument resolution that does not adequately capture the spectrally narrow contributions of stratospheric O_2_. Resolution improvements to better than 100 MHz are anticipated to improve fit qualities. Importantly, residuals are smaller away from the line center due to contributions from higher pressure (lower altitude) oxygen, and this is the atmospheric region of most relevance in GHG metrology.

### 3.2. Water Mixing Ratio Retrieval Procedure

**Figure 7:**
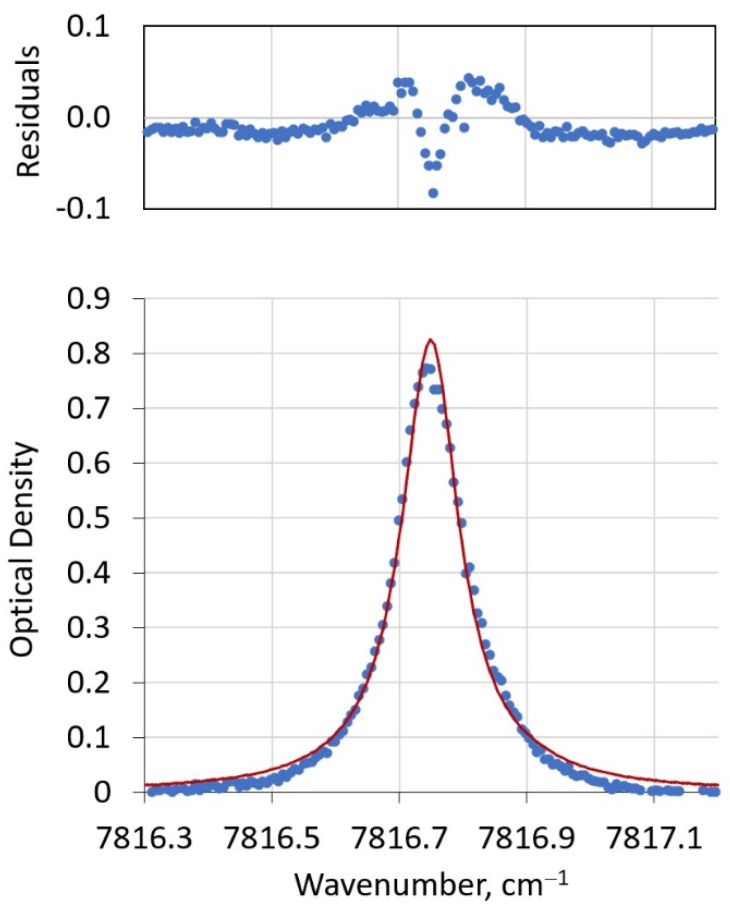
Lower panel: representative fit of PHOCS water feature. The upper panel shows the residuals of the fit.

The fitting of the water feature at 7816.75 cm^−1^ proceeds in an analogous fashion with a few notable differences. The temperature and pressure polynomial coefficients from the fitting of the oxygen feature define their vertical profiles needed to retrieve pre-calculated water absorption coefficients throughout the sun path. The initial guesses for the water mixing ratio in each level are drawn from the mean of the binned radiosonde data for each 1 km level. For this part of the retrieval, the integrated coefficients for each level need only be calculated once, saving considerable computational time in the remainder of the retrieval. As before, a Nelder–Mead optimization is performed, but now with considerably more variables (the water mixing ratio within each 1 km bin). As was done for fitting the oxygen feature, the fits were guided by a weighting factor determined by both mean and standard deviations of each bin’s radiosonde data (Equation (1)). A fit of the water feature in the same spectral sweep shown in Figure 6 appears in Figure 7.

In addition to the advantage of only calculating the vertical profile of absorption coefficients once, it should be noted that fitting this large number of parameters (and thus increasing vertical resolution) is enabled by two additional factors. First, the relatively large spectral width of the water feature and the high spectral resolution of the PHOCS instrument provide substantial oversampling for the fit (in the data shown in Figure 7, this factor is approximately 10:1). A second factor is the wealth of historic radiosonde data available, particularly for low altitudes where water has the highest mixing ratio and is the most variable. It should be noted that no adjustment to the wavenumber calibration was performed for this fit. It is not clear if deviations of modeled and observed spectra, particularly at frequencies away from the line center, suggest a need for better instrument calibration and/or improved spectral modeling parameters. For future generations of the PHOCS instrument, we are exploring more precise calibrations using a Fabry–Perot etalon.

## 4. Discussion

### 4.1. Comparison of RS Temperature Profiles to MERRA-2

**Figure 8:**
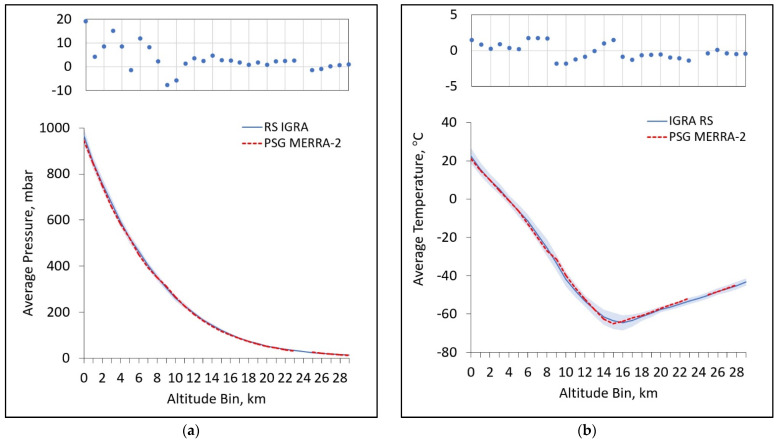
A comparison of the average (**a**) pressure vertical profile and (**b**) average temperature vertical profile for our decade-long, 7-day window with standard deviations shown as a blue shaded region and MERRA-2 reanalysis temperature profiles as red dashed lines. Note that MERRA-2 data were only extracted over a decade-long, 1-day window for 29 July 2010–2020. Residuals in the top panels of each figure show the difference between average IGRA values and average MERRA-2 values.

As starting estimates for the pressure and temperature profiles, our previous work relied on the MERRA-2 database, which is largely based on satellite measurements. Retrievals were obtained via NASA’s PSG tool [24]. However, these profiles are generally not available for several days or more before the present, which obviously limits the use of MERRA data for real-time measurements. There is good agreement between MERRA reanalysis data and RS vertical profiles, as shown in Figure 8, and the latter provides near-real time, locally derived data with greater vertical resolution near the surface. This makes it a better tool when constraining surface fluxes and narrowing down potential sources of trace gases. Its most obvious limitation is that as the radiosonde package rises through the atmosphere, the radiosonde balloon stretches from its starting diameter of about 1.5 m to its limit at 6–8 m after which it bursts, typically in the stratosphere, at or below 35 km [25].

### 4.2. Comparison of RS Temperature Profiles to Standard and Reference Atmospheres

**Figure 9:**
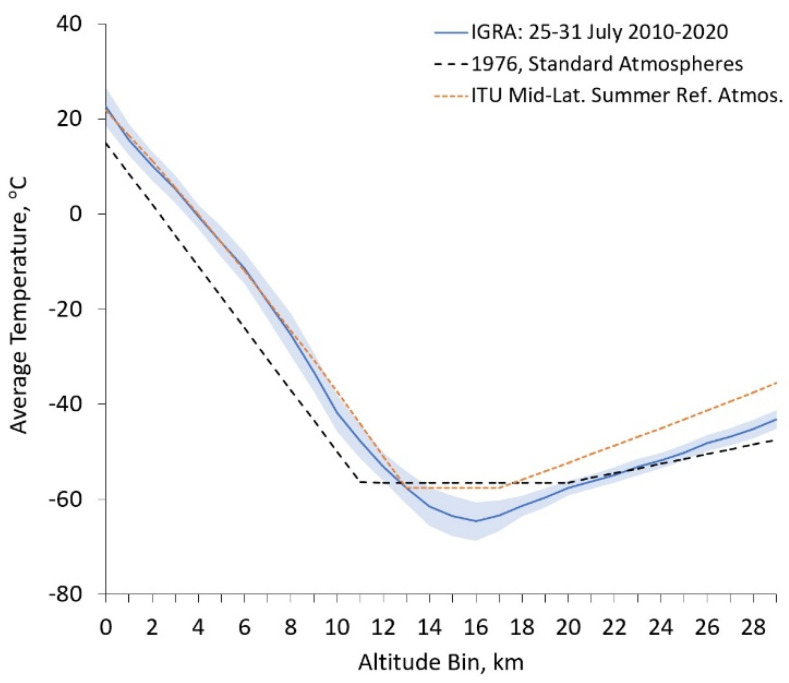
A comparison of the average temperature vertical profile obtained from IGRA for our decade-long, 7-day window with standard deviations shown as a blue shaded region, 1976 U.S. standard atmospheres (black dashed line), and the ITU Radiocommunication Assembly mid-latitude summer reference atmosphere (orange dotted line).

The 1976 U.S. standard atmospheres are still in use today for an idealized, steady-state view of Earth’s atmosphere at mid-latitudes [26]. Another source for a reference atmosphere is that used by the ITU Radiocommunication Assembly, which recommends the use of the Summer Reference Atmosphere for mid-latitudes [27]. They state that if more reliable local data are available, they should be used instead. The reason for this becomes apparent when looking at Figure 9. RS data, particularly for temperature and water vapor vertical profiling, provide a more continuous profile with greater vertical resolution. Furthermore, the use of more accurate vertical profiles as initial estimates in the retrieval algorithm allows for more rapid convergence in the fitting process. Profiles for the U.S. standard atmospheres and ITU mid-latitude summer reference atmosphere were created using their respective provided equations and altitude guidelines for tropopause height.

### 4.3. On-Going Retreival Improvements: Bayesian Inference for Vertical Mixing Ratio Determinations

The Bayesian paradigm applies prior knowledge and observations to a model being tested. It is the foundation upon which inverse modeling in the atmospheric sciences is built [28]. The variance of the prior distribution is expressed, which leads to posterior probability distributions that inform subsequent measurements and help quantify the precision. Our next generation PHOCS instrument will be deployed in a permanent installation at the Smithsonian Environmental Research Center’s Global Change Research wetland (https://serc.si.edu/gcrew (accessed on 5 August 2021)) over the 3rd–4th quarters of 2021. This instrument will use three laser heterodyne units to detect oxygen, water, carbon dioxide, and methane. With the enormous data record expected from this installation, we plan to incorporate observed data into the determination of posterior distributions, which will provide feedback to the weighting of subsequent fits and quantify uncertainty in real-time measurements.

## 5. Conclusions

An analysis of historic pressure and temperature profiles from radiosonde launches for use as Bayesian priors in the fitting of molecular oxygen line shapes has been presented. When decadal data are averaged over one-week windows, it is found that the distributions for both temperature and pressure are relatively narrow, with temperature displaying the greatest variation closest to the surface. Even for these data, it was observed that the full span of data fell within three standard deviations of the mean for each altitude (indicating that there are few outliers). We argue that this historic RS record produces superior temperature and profiles to standard atmosphere characterizations, and is consistent with retrospective analyses such as MERRA-2, but is advantaged by its utility for real-time analysis of LHR data using vertical profiles with greater vertical resolution. Further, careful fitting of oxygen spectral features may be used to refine these profiles and to identify outlying conditions such as near-surface inversions. Together, this approach extends the utility of oxygen vertical profiling beyond wet air corrections. Current instrument goals include the improvement of the current 200 MHz resolution to 100 MHz or better, which would lead to a reduction in residuals near the line center and a better overall fit of the O_2_ spectral line. However, even for the current oxygen measurements, the errors in fitting are largely attributable to the low-pressure contributions of stratospheric absorption near the line center. Although broadly important in atmospheric science, our goal here is to develop a tool to inform tropospheric greenhouse gas measurements. Residuals away from the oxygen line center, from higher pressure and temperature regions of the atmosphere, are quite small. To that end, we demonstrated the use of PHOCS-derived, temperature and pressure vertical profiles in the real-time determination of greenhouse gas mixing ratios.
